# Mr. Shearly's Case of Gout

**Published:** 1805-08-01

**Authors:** William Shearly

**Affiliations:** Member of the Royal College of Surgeons, London. Royal Naval Hospital, Deal


### *To the Editors of the Medical and Physical Journal.*

GENTLEMEN,

AS the late plan of treatment recommended so strongly by Dr. Kinglake for the cure of gout, has excited the public attention for some considerable time, I was determined, if ever a case came under my care while I remained in hospital practice, I would give it a fair trial. At length one has presented itself, in which I am happy to say the application of cold proved successful in arresting the progress of this truly harrassing and painful complaint. The reader will observe I have not entered into the labyrinth of hypothetical reasoning, but have confined myself entirely to the relation of symptoms which existed at the time I began this plan of treatment, and those which arose during the process of the cure.

James Cobb, was admitted into the Royal Naval Hospital, June 12, 1805. There appeared great inflammation in both feet, accompanied with considerable tension. The account which he himself gave relative to the complaint was the following. About two o'clock on the morning of the 7th he was awoke by a violent pain situated in the ball of the great toe of the right foot, attended with great inflammation, flammation, which extended towards the external malleolus of the same foot. This inflammation increased considerably during the day, accompanied with great tension. He had no interval of ease during the following day, and his left foot was attacked on the following morning with the same symptoms exactly that the right one had been attacked with on the preceding. A large quantity of opium was administered by the surgeon of the ship, but without procuring the least ease. He had been attacked twice before, exactly in a similar manner, two years intervening from each attack. During the latter exacerbation he had been attended by an eminent physician, who advised flannels and other warm applications to be applied to the feet, with the addition of stimulating medicines, joined with opium internally. These applications had not the desired effect, as the patient was laid up for seven weeks, without deriving the least benefit. I considered this as a true paroxysm of gout, consequently as a proper case for applying cold, locally. I therefore ordered a pail of cold water to be placed near his bed, and cloths dipped in this to be applied to the feet, and to be renewed every ten minutes, This plan of treatment was begun as soon as the patient was placed comfortably in bed, which took place about six o'clock in the evening. During my last visit, at ten o'clock, I found him in a complete diaphoresis, and his pulse accelerated to 140, extremely full, with great inclination to sleep. This last symptom I consider as totally unconnected with my local applications, it being produced in consequence of his taking an opiate. He pursued this plan of treatment during the night, and through the whole course of the following day and night. At ten o'clock on Saturday morning he sent for me (according to my request) and complained of great pain about the epigastric region. He told me he could not compare the pain to any thing else but as if he had swallowed a portion of any boiling liquid. He also complained of a slight pain situated near the middle of the occipital bone. I ordered him to desist immediately from persevering in the cold applications, and gave him the following medicine: ℞. M. camp. ℥vj. tinct. card. c. ℥ij. ammon ppt. ʒß. M. ft. mist. capt. coch. iij. secunda qq. hora. Sumatur etiam vin. rub. liberaliter. The patient had not taken three doses of this medicine before he found himself perfectly easy and composed. At the time the cold was taken away, the tension, pain, and inflammation, had entirely disappeared in both feet, as not the least trace of this last symptom could could possibly be detected. He persevered in the use of the medicine and wine; the latter he took to the amount of a pint and a half in the course of twenty-four hours. He now began to eat his food with an appetite, and was able to walk about the ward with the assistance of crutches, on the fifth day after his admission. Towards evening he complained of pain in the right foot, which was unattended with inflammation. I ordered cold applications to be resorted to, and the patient to be confined to his bed. This was continued about twelve hours, when he informed me the pain had entirely subsided. Since this period, which is about three weeks, he has never had one disagreeable symptom, only complaining of a little stiffness about the instep of each foot.

I am, &c.

WILLIAM SHEARLY,

Member of the Royal College of Surgeons, London.

*Royal Naval Hospital, Deal, July* 6, 1805.

